# Looking “Cherry Red Spot Myoclonus” in the Eyes: Clinical Phenotype, Treatment Response, and Eye Movements in Sialidosis Type 1

**DOI:** 10.5334/tohm.652

**Published:** 2021-12-09

**Authors:** Giulietta M. Riboldi, John Martone, John-Ross Rizzo, Todd E. Hudson, Janet C. Rucker, Steven J. Frucht

**Affiliations:** 1 Fresco Institute for Parkinson’s and Movement Disorders, Department of Neurology, New York University School of Medicine, NY, US; 2 Department of Neurology, New York University School of Medicine, NY, US; 3 Department of Physical Medicine and Rehabilitation, New York University School of Medicine, NY, US; 4 Department of Ophthalmology, New York University School of Medicine, NY, US

**Keywords:** sialidosis type 1, myoclonus, eye movements, sodium oxybate

## Abstract

Sialidosis type 1 is a rare lysosomal storage disorder caused by mutations of the neuraminidase gene. Specific features suggesting this condition include myoclonus, ataxia and macular cherry-red spots. However, phenotypic variability exists. Here, we present detailed clinical and video description of three patients with this rare condition. We also provide an in-depth characterization of eye movement abnormalities, as an additional tool to investigate pathophysiological mechanisms and to facilitate diagnosis. In our patients, despite phenotypic differences, eye movement deficits largely localized to the cerebellum.

## Introduction

Sialidosis, also known as mucolipidosis type 1, is a rare autosomal recessive lysosomal storage disorder caused by mutations in the neuraminidase (*NEU1*) gene. More than 40 pathogenic mutations of *NEU1* have been reported [1]. Different mutations induce variable degrees of residual neuraminidase function, which affect the age of disease onset and symptom severity. The disease has been categorized into sialidosis type 1, presenting with a milder phenotype and a later age of onset, and sialidosis type 2, in which disease starts earlier in life and causes severe systemic symptoms (such as hydrops, hepatosplenomegaly, dysostosis multiplex, coarse facial features, renal and cardiac involvement, and ophthalmological abnormalities such as cherry red spots, corneal clouding, cataracts, nystagmus, and strabismus).

The incidence of sialidosis is 1 in 4.2 million people per live births [2]. The onset of sialidosis type 1 is typically in the second or third decade of life. “Cherry red spot myoclonus” is often used to refer to type 1 as these two signs are classically present, together with ataxia, hyperreflexia and loss of vision. Progressive myoclonic epilepsy can also be present. Patients affected with sialidosis type 1 can manifest with resting or action myoclonus, which can be sensitive to tactile and auditory stimuli; light sensitivity is usually more indicative of other forms of progressive myoclonic epilepsy, such as Gaucher’s disease, Lafora disease, or ceroid-lipofuscinosis [3]. The myoclonus can present with an appendicular, as well as axial, distribution. The diagnosis of sialidosis is confirmed by the presence of pathogenic mutations in the *NEU1* gene and reduced neuraminidase activity in cultured fibroblasts and leucocytes.

Because of great variability in disease symptoms and signs, particularly of the myoclonus, patients affected with sialidosis type 1 can appear very phenotypically different from one another. In this case series, we present three patients with sialidosis type 1, all differing in their disease severity and patterns of myoclonus (***Table 1***). Moreover, we also performed detailed quantitative eye movement analyses. To our knowledge, detailed eye movement assessments have not been previously reported in patients with sialidosis type 1.

**Table 1:** **Characterization of the clinical and genetic features of the described cases**. Demographic, genetic and phenotypical characterization, as well clinical and medication history of the described cases are reported in the table. OD: oculus dexter (right ye); OS: oculus sinister (left eye).


	AGE OF ONSET	AGE AT DIAGNOSIS	CURRENT AGE	*NEU1* GENETIC MUTATION	PRESENTING SYMPTOMS	PROGRESSION	CURRENT TREATMENT

**CASE 1**	7 years	8 years	19 years	c.727G>A (p.G243R) + c.544A>C (p.S182R)	– Poor vision – 20/400 OD and OS –“cherry red spot” at ophthalmological evaluation	–**Vision loss** **Small fiber neuropathy** **Lower limb** more than upper limbs **action myoclonus** since the age of 13 years, mild wide based gait –**Depression** and **attempted** suicide	Trihexyphenidyl à clonazepam, levetiracetam

**CASE 2**	8 years	32 years	40 years	homozygous c.629C>T, (p.P210L)	–Loss of vision – 20/400 both eyes –“cherry red spot” at ophthalmological evaluation	–At the age of 14 years he developed **action myoclonus** of the upper limbs **Balance** impairment and wide based gait –**Vocal** myoclonus and myoclonus of the lower cranial region	Valproic acid à levetiracetam, perampanel à clonazepam, zonisamide. Sodium oxybate was attempted

**CASE 3**	School age	12 years	31 years	c.644T>C (p.L215P) + c.649G>A (p.V217M)	–Difficulties seeing the blackboard (20/30 OD, 20/40 OS) – “cherry red spot” at ophthalmological evaluation	–Worsening of vision and color misperception –**Gait ataxia, slurred speech** and **postural, action and stimulus sensitive myoclonus** of the upper limbs since the age of 20 years –Generalized **tonic-clonic seizures**	Clonazepam/levetiracetam Sodium oxybate was attempted


## Methods

### Clinical assessments

Patients were seen and assessed at NYU Langone Health in the Department of Neurology (Marlene and Paolo Fresco Institute for Parkinson’s and Movement Disorders and the Ocular Motor Laboratory). Clinical history and examination were collected by Movement Disorder specialists. Patients were video recorded during the Movement Disorder visits after appropriate consent was obtained.

#### Eye movement analysis methods

A detailed clinical eye movement examination was performed. Binocular horizontal and vertical eye movements were recorded with infrared oculography (EyeLink 1000 Plus, SR Research, Mississauga, Ontario, Canada) (sampling frequency 500 Hz, spatial accuracy 0.5 degrees), following a 13-point spatial calibration. The head was stabilized in a forehead cradle. The visual stimulus for saccades was a solid white circle displayed on a computer screen with a dark gray background. Gaze stability during visual fixation was assessed centrally and eccentrically at ±18.5 degrees horizontally and ±11 degrees vertically. Saccades were tested to target jumps through a range of amplitudes up to ±18 degrees horizontally and ±11 degrees vertically. Horizontal antisaccade error rates were assessed, with instructions to look in the mirror location, horizontally, of the target displacement relative to screen center. Smooth pursuit was assessed with the visual target moving sinusoidally at 0.25 Hz ±10 degrees horizontally and vertically. Eye movement data were analyzed off-line using custom Matlab software. Data recorded within 100 ms of a blink were automatically eliminated. Saccades were identified via an adaptive threshold mechanism and velocities and accelerations were computed from position traces using a low-pass differentiator.

## Results

### Patient 1

#### Clinical presentation

A 19-year-old Caucasian woman was diagnosed with sialidosis type 1 at the age of 8. When she was 7 years old, she began to develop worsening vision. Ophthalmological evaluation revealed bilateral cherry red spots. The diagnosis was confirmed after skin biopsies showed a reduction of neuraminidase activity. Genetic testing for *NEU1* gene revealed a heterozygous pathogenic variant (c.727G>A, p.G243R), and a new missense variant c.544A>C (p.S182R), not found in large control databases (ExAC/gnomAD, dbSNP). Levels of oligosaccharides were increased although lower than expected, consistent with her milder phenotype. Her mother recalled that, as a child, she had frequent ENT infections but normal psychomotor development.

During the course of her disease, her vision loss significantly progressed, with a prominent decline around age 17, which was accompanied by the onset of oscillopsia and awareness of shaking eyes. She noted day to day variability in the oscillopsia and eye shaking. She was also diagnosed with a small fiber neuropathy characterized by pain and burning sensation of her hands and feet. She was treated with pregabalin and symptoms almost totally resolved after six years. A few years after her initial diagnosis, she developed severe depression with suicide attempts, treated with sertraline and escitalopram.

Around the age of 13 she developed jerks in her lower limbs while asleep, as noticed by her mother. Recent exam revealed visual acuity of 20/400 in each eye; prominent myoclonus of her lower extremities triggered by walking and causing multiple falls; mild action myoclonus of the left more than the right arm; and action myoclonus of the legs while standing, with both a positive and a negative component. These symptoms were significantly improved by the light touch of a wall or a supportive person. Her gait was moderately wide-based (***Video 1***, Segment 1).

**Video 1:** **Neurological examination of case 1–3. Segment 1:** examination of case 1 shows mild myoclonus in the upper limbs, especially at target and with action, and cerebellar signs such as overshooting; gait was wide based and it presented frequent brakes due to myoclonic jerks in the lower limbs especially upon turning. **Segment 2:** case 2 was evaluated before and 45 minutes after the administration of Sodium Oxybate 1 gr. The two recording are presented side-to-side to help appreciating the improvement after the administration of the medication. At baseline, patient presented frequent volleys of resting, postural and action myoclonus in the upper limbs, especially at target, which significant impact on finalized activities (such as pouring water from a cup). After the administration of Sodium Oxybate 1 gr, postural and action myoclonus improved, allowing faster and smoother execution of different tasks, such as pouring water. **Segment 3:** postural and action myoclonus are noticeable in the upper limbs, mostly noticeable at target and with different tasks (i.e. drawing spirals and pouring water). Stimulus sensitive myoclonus to pin was present. Enhanced startle to unexpected sounds when walking. Tandem gait was affected.

She was previously treated with trihexyphenidyl, with no benefit, and clonazepam up to 3 mg daily with only partial improvement. Recently, levetiracetam was attempted. Her symptoms were noted to be alcohol responsive. Shortly after diagnosis, she participated for a short time in a clinical trial with miglustat without benefit. Recent brain MRI showed only small caliber optic nerves and chiasm.

#### Eye movement analysis

Ocular motor examination revealed a complex pattern of variable abnormal spontaneous eye movements in central gaze; square wave jerks in central gaze alternated with nystagmus (at times with a pendular waveform, at other times with a jerk waveform) in central gaze (***Video 2***). At other times, fixation was fairly stable in central gaze. During the epochs of nystagmus in central gaze, nystagmus was also visualized in eccentric gaze without a null zone (i.e., there was no gaze position in which nystagmus was dampened). These eye movement findings in central gaze were also variable across visits, nearly absent on some days and prominent on others. This complex and variable ocular motor pattern in central gaze in this patient may have arisen secondary to substantial central vision loss. Saccadic intrusions were superimposed on smooth pursuit; and saccadic hypermetria was present. Eye movement recordings at visit one (the patient verbally reported being able to see the visual target during recordings; data quality was good, though accuracy likely degraded to some extent by her reduced visual acuity) demonstrated marked difficulty with following saccadic targets, with excess erratic saccades poorly related to target jumps (**Supplementary Figure 1A**), saccadic hypermetria (**Supplementary Figure 1A**), macro-square wave jerks (**Supplementary Figure 1A**), and saccadic smooth pursuit (**Supplementary Figure 1B**). Saccade speed was preserved in both the horizontal and the vertical plane as demonstrated by the normal main sequence relationships between horizontal saccade amplitude and peak velocity (**Supplementary Figure 1C**). Eye movement recordings at visit two captured the variability of eye movements in central position that were variable day to day (***Video 2***).

**Video 2:** **Eye movement assessments**. Narrated video demonstrating clinically visible eye movement abnormalities in all 3 patients.

### Patient 2

#### Clinical presentation

A 40-year-old man from Ecuador was diagnosed with sialidosis type 1 at the age of 38. He was born prematurely and jaundiced, but had normal motor and cognitive development. At the age of 8, he started to wear glasses because of progressive vision loss. At the age of 14, he developed involuntary movements, chiefly affecting his hands during action. Three years later his balance started to worsen as well. Myoclonus significantly impacted his daily life. He was initially treated with valproic acid 500 mg, although he never had a seizure. He also tried levetiracetam and perampanel, which he did not tolerate. Eventually he started clonazepam (1 mg twice daily) and zonisamide (300 mg daily). Since myoclonus was very alcohol responsive sodium oxybate was attempted with clear improvement of his symptoms (***Video 1***, Segment 2). Unfortunately, the medication was discontinued due to the emergence of visual hallucinations.

His past medical history was notable for anemia requiring yearly transfusions, and osteosclerosis of the left shoulder due to an accident at the age of 15 years. Visual acuity was 20/400 in both eyes and cherry red maculae were identified on ophthalmological evaluations. Because of the marked visual impairment, he required the use of a white cane to walk.

At the age of 38, confirmatory genetic testing revealing a homozygous missense variant c.629C>T, p.Pro210Leu, in the *NEU1* gene, previously not reported in patients with sialidosis nor found in large control databases (ExAC/gnomAD, dbSNP). Sialidase activity was absent in fibroblasts while β-Galactosidase activity was preserved, corroborating the genetic diagnosis. Recent examination revealed very prominent myoclonus at rest and with action with both positive and negative jerks. He had vocal myoclonus with paroxysms in the lower cranial region triggered by speaking and animation. Action myoclonus was particularly severe, with an exacerbation of the myoclonus with fine action such as copying a spiral or writing (***Video 1***, Segment 2). When he performed the same activity with his eyes closed, myoclonus improved. There was no overt appendicular dysmetria. Gait was narrow-based, Romberg was absent, but standing for a prolonged period provoked myoclonic volleys.

#### Eye movement analysis

Ocular motor examination revealed subtle downbeat nystagmus on lateral gaze and subtle gaze-evoked nystagmus characterized by a few beats of upbeat on far upgaze; saccadic hypermetria was present as well as saccadic smooth pursuit (***Video 2***). Eye movement recordings were attempted but no useful data were obtained due to his poor visual acuity.

### Patient 3

#### Clinical presentation

**Figure 1:**
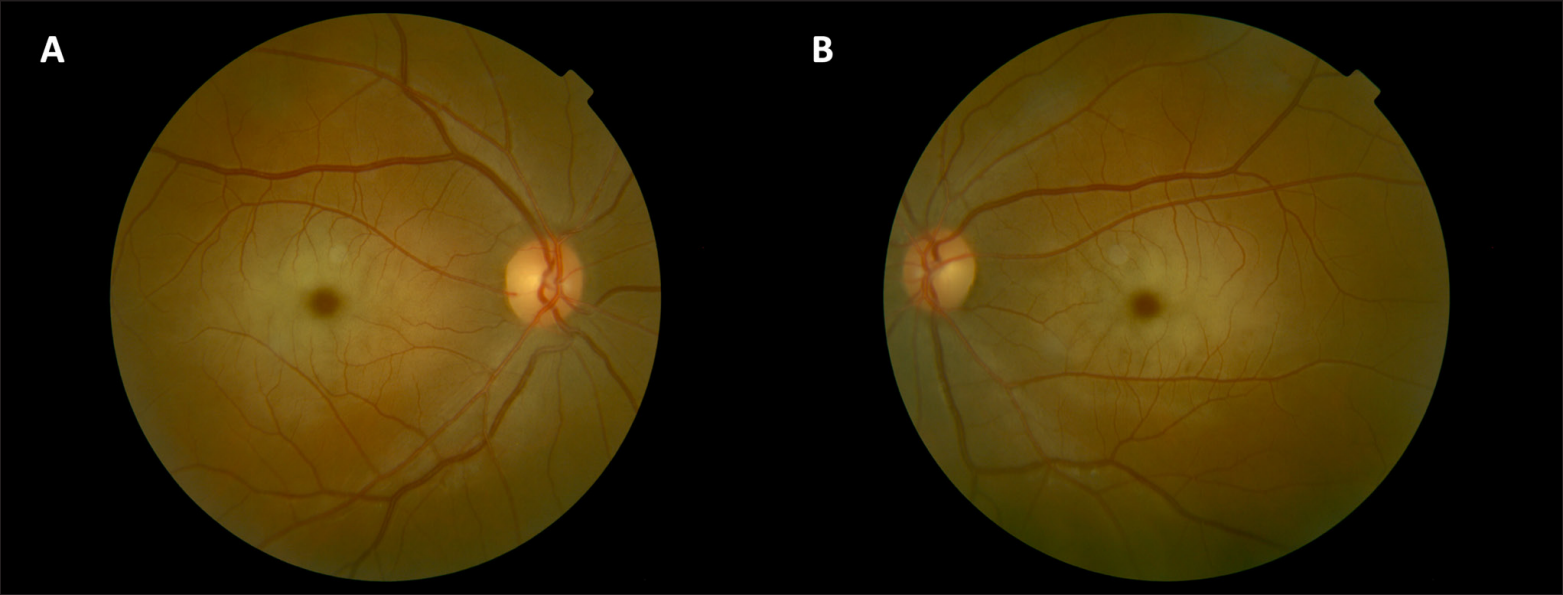
Fundus photographs of the right **(A)** and left **(B)** eyes of Case 3 demonstrating bilateral cherry red spots.

A 31-year-old man of Haitian heritage was diagnosed with sialidosis type 1 at the age of 12. There was no consanguinity in the family. He came to medical attention after developing difficulty seeing the board at school. Ophthalmological evaluation revealed bilateral macular cherry red spots (***Figure 1***). Sialidosis type 1 was genetically confirmed by the findings of two variants in the *NEU1* gene: c.649G>A (p.V217M), a previously reported pathogenic variant, and c.644T>C (p.L215P), a variant never previously reported in patients with sialidosis type 1 and not found in large databases of unaffected subjects (ExAC/gnomAD, dbSNP).

Following the diagnosis, he had progressive worsening of his vision and color perception. This was particularly true of his distance vision, especially at night. Around the age of 20 he started to develop mild gait ataxia, slurred speech and myoclonus, mostly affecting his upper limbs. Clonazepam was started with benefit. Due to four generalized tonic-clonic seizures, levetiracetam was added with improvement in myoclonus. However, his symptoms were still debilitating and significantly interfering with his daily activities. His symptoms were exquisitely alcohol responsive. Sodium oxybate was started with great improvement and only residual very mild postural and action myoclonus of his hands. Stimulus-sensitive myoclonus disappeared after starting the medication. Gait ataxia remained mild (***Video 1***, Segment 3).

Brain MRI showed reduced size of the cerebellar vermis and brain MRI spectroscopy revealed elevated creatinine in the superior cerebellar vermis. EMG was consistent with a distal motor neuropathy. High frequency hearing loss was detected on auditory tests.

#### Eye movement analysis

**Figure 2:**
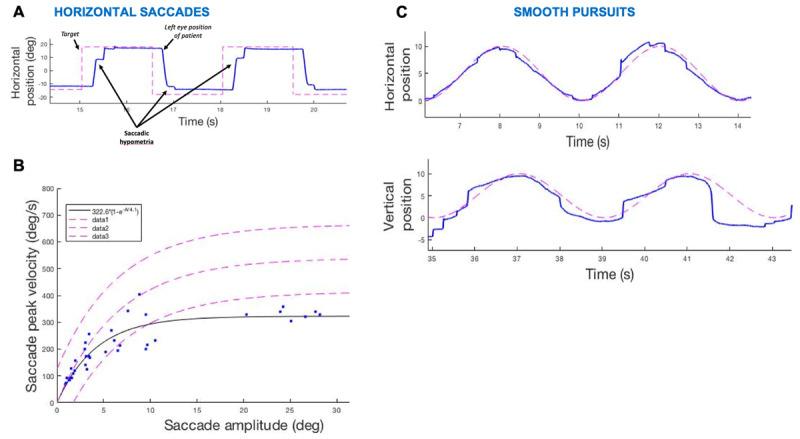
**Eye movement recording of Case 3. A)** Saccadic hypometria (undershoots). **B)** Slow horizontal saccades in main sequence. **C)** Saccadic smooth pursuit (upper figure – horizontal, and lower figure – vertical).

Ocular motor examination revealed saccadic horizontal greater than vertical smooth pursuit gain, intermittent horizontal and vertical saccadic hypometria, and prolonged vertical saccade latency. Optokinetic nystagmus was normal horizontally, vertically only slow phases were present (***Video 2***) suggesting a possible early saccadic gaze palsy. In addition, there was mild right sixth nerve paresis and convergence insufficiency. Eye movement recordings confirmed saccadic hypometria (***Figure 2A***), saccadic slowing horizontally (***Figure 2B***) and vertically (confirming a saccadic gaze palsy, most likely from brainstem burst neuron involvement), and saccadic horizontal and vertical smooth pursuit (***Figure 2C***).

## Discussion

Sialidosis type 1 is a rare, recessive condition. A recent extensive review of the literature revealed descriptions of 45 cases with a genetically confirmed diagnosis of sialidosis type 1 [4]. Deficiency of the neuraminidase enzyme, caused by pathogenic variants of the *NEU1* gene, results in impaired metabolism of the sialic acid on the oligosaccharides and glycoproteins. Sialic acid–rich macromolecules accumulate in the cytoplasm of cells in different target tissues as lipofuscin rich inclusions, resulting in cellular dysfunction and disease manifestations with variable degrees of severity according to the residual activity of the enzyme [5].

The classic presentation of sialidosis type 1 is characterized by a combination of myoclonus, ataxia and seizures, with variable visual complaints. Although cases with an earlier age of onset more frequently have a classical presentation, not every patient presents the full disease spectrum [4]. As a consequence, diagnosis of this already rare condition is made even more difficult, especially when clinicians are not familiar with its presentation. A delayed diagnosis presents a burden for patients and their families. Our aim in reporting these three well-characterized patients with sialidosis type 1 is to document in detail phenotypic variability and responses to treatment, as well as to increase awareness of this condition amongst clinicians. In particular, we outlined the variability of the cortical myoclonus: the first patient had lower limb predominant myoclonic jerks, the second presented a prominent vocal and lower cranial myoclonus significantly affecting his speech, and the third had upper limb predominant myoclonus. In all three cases, myoclonus was significantly triggered by action, but present at rest as well. As expected, myoclonus was alcohol-responsive in these patients, an important consideration given its therapeutic implications. Traditional approaches with antiepileptic drugs and benzodiazepines, usually to be used in combination, can be beneficial in these patients [6]. However, when symptoms are resistant to first-line approaches sodium oxybate (SBX) can be considered, as well. SBX, a sodium salt of γ-hydroxybutyrate, has been traditionally used and approved for cataplexy in narcolepsy. However, this medication has been proven to be beneficial in alcohol-responsive movement disorders, such as tremor, post-hypoxic myoclonus, myoclonus dystonia, and spasmodic dysphonia [7,8,9,10,11]. In all of our patients, this treatment significantly improved quality of life, though one had to discontinue use due to side effects.

In our cases additional features, such as polyneuropathy (patient 1) and sensorineural hearing loss (patient 3), described in only a few cases in the literature, were found [12,13]. It is currently unclear if these are concomitant findings unrelated to sialidosis or if they represent additional manifestations of this disease. So far homozygous or compound heterozygous missense, frameshift, truncating mutations as well as duplications and deletions of the *NEU1* gene have been reported in patients with sialidosis type 1 [4]. Biochemical findings, such as decrease in α-neuraminidase activity on skin biopsy or increased oligosaccharides in the urine, can further support the diagnosis in case of novel or not clearly pathogenic genetic variants, such as in case 2 in this work [14].

Detailed ocular motor assessments were performed in our patients, as eye movement abnormalities can be very informative in movement disorders. Indeed, they can reflect the distribution and severity of affected systems. Eye movements have, to the authors’ knowledge, never been systematically studied in patients with sialidosis type 1. Previous reports focus on the afferent abnormalities and vision loss resulting from the well-recognized pathological storage product accumulations in the ganglionic cells of the retina (resulting in the classic “cherry red spot”, present in all of our patients), optic nerve atrophy as documented by optical coherence tomography (OCT), and cataracts [15,16]. The classic cherry red spot is not found exclusively in sialidosis and in rare cases of sialidosis type 1 it may also be absent [17,18]. This finding reflects the sparing of the central area of the retina in contrast to the opacification of the surrounding ganglion cells of the retina that can be caused by a variety of conditions, such as accumulation of storage material in other Lysosomal storage diseases (i.e. Niemann-Pick disease, Tay-Sachs disease, Galactosialidosis, Farber lipogranulomatosis, Metachromatic leukodystrophy), or traumatic or vascular retinal ischemia [18].

Eye movement analysis (clinical examination followed by quantitative eye movement recording to enhance detection of subtle pathology) in our patients showed several abnormalities. Before discussing those abnormalities likely directly due to ocular motor effects of sialidosis, special mention of the variable ocular motor patterns in central gaze in patient 1 is required. This patient demonstrated minute-to-minute and day-to-day variability in central gaze, at times demonstrating stable fixation, square wave and macro square wave jerks, or nystagmus (with both pendular and jerk components) [19,20]. Given this high degree of variability, two possibilities exist: abnormal fixational eye movements as a secondary consequence of severe central vision loss or voluntary control of congenital nystagmus leading to intermittent pendular nystagmus. The former is more likely, given the patient’s awareness of the development of oscillopsia and shaking eyes at a time of a marked decline in her vision. Many of the other eye movement findings in this group of patients are consistent with cerebellar involvement, including saccadic smooth pursuit, saccadic hypometria or hypermetria, downbeat nystagmus, and gaze-evoked nystagmus. In one patient (patient 3), loss of the fast phases of optokinetic nystagmus and slowing of saccades suggested involvement of saccadic burst neurons in the brainstem. This patient alone also had mild right sixth nerve paresis and convergence insufficiency. Though the eye movement abnormalities seen in these patients are non-specific and variable, their presence in combination with myoclonus may suggest sialidosis type 1 and they certainly suggest that further detailed ocular motor phenotyping is needed in this disease. Moreover, in the context of possible new therapeutic approaches, they can represent an additional and easily accessible outcome measure for the evaluation of disease progression and treatment response, as parameters such as smooth pursuit gains, the size and extent of saccadic dysmetria and saccadic intrusions, and nystagmus time constants can be easily and precisely quantified.

## Additional File

The additional file for this article can be found as follows:

10.5334/tohm.652.s1 Supplementary Figure 1. Eye movement recording of Case 1. A) Marked difficulty following saccadic targets with excess erratic saccades not in relation to target jumps, saccadic hypermetria (overshoots), and macro square wave jerks. B) Saccadic smooth pursuit. C) Main sequence relationships between horizontal saccade amplitude and peak velocity are normal.
