# Supplementary material for: Looking “Cherry Red Spot Myoclonus” in the Eyes: Clinical Phenotype, Treatment Response, and Eye Movements in Sialidosis Type 1
Source: Tremor Other Hyperkinet Mov (N Y). 2021 Dec 9;11:53. doi: 10.5334/tohm.652 (PMC8681143; doi:10.5334/tohm.652)
Supplement: Supplementary Figure 1. — Eye movement recording of Case 1. A) Marked difficulty following saccadic targets with excess erratic saccades not in relation to target jumps, saccadic hypermetria (overshoots), and macro square wave jerks. B) Saccadic smooth pursuit. C) Main sequence relationships between horizontal saccade amplitude and peak velocity are normal. [file tohm-11-1-652-s1.pdf]

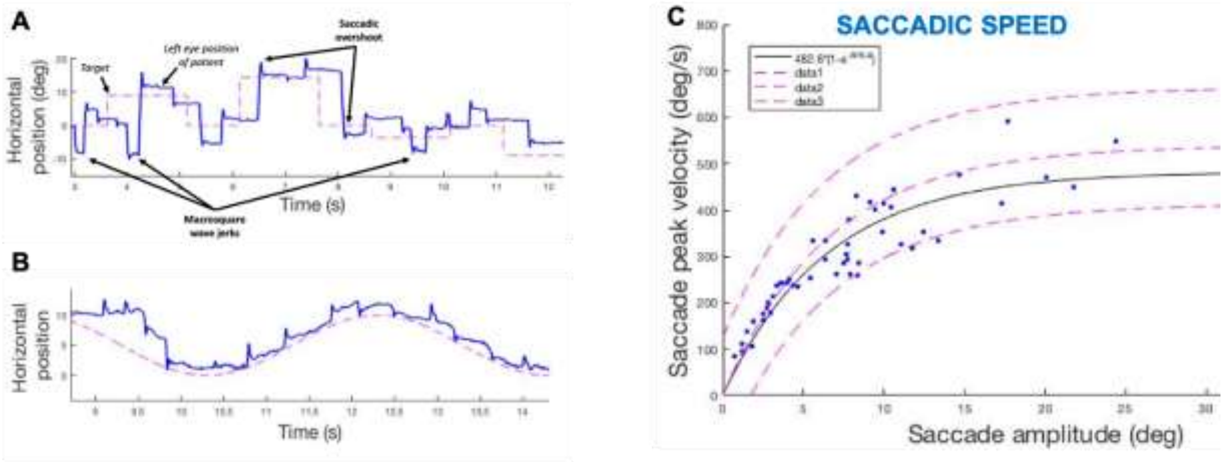


**Supplementary Figure 1. Eye movement recording of Case 1.** A) Marked difficulty following saccadic targets with excess erratic saccades not in relation to target jumps, saccadic hypermetria (overshoots), and macro square wave jerks. B) Saccadic smooth pursuit. C) Main sequence relationships between horizontal saccade amplitude and peak velocity are normal.
